# Excretory-secretory products from the brown stomach worm, *Teladorsagia circumcincta*, exert antimicrobial activity in in vitro growth assays

**DOI:** 10.1186/s13071-022-05443-z

**Published:** 2022-10-02

**Authors:** James Rooney, Timothy L. Williams, Holly M. Northcote, Fiona E. Karet Frankl, Daniel R. G. Price, Alasdair J. Nisbet, Russell M. Morphew, Cinzia Cantacessi

**Affiliations:** 1 Department of Veterinary Medicine, University of Cambridge, Cambridge, CB3 0ES UK; 2 Institute of Biological, Environmental and Rural Sciences, Aberystwyth University, Aberystwyth, SY23 2DA UK; 3 Department of Medical Genetics, University of Cambridge, Cambridge, CB2 0QQ UK; 4 Vaccines and Diagnostics Department, Moredun Research Institute, Penicuik, EH26 0PZ UK

**Keywords:** *Teladorsagia circumcincta*, Gastrointestinal helminth, Ruminant, Microbiome, Extracellular vesicle, Antimicrobial peptide, Excretory-secretory products

## Abstract

**Background:**

Over the past decade, evidence has emerged of the ability of gastrointestinal (GI) helminth parasites to alter the composition of the host gut microbiome; however, the mechanism(s) underpinning such interactions remain unclear. In the current study, we (i) undertake proteomic analyses of the excretory-secretory products (ESPs), including secreted extracellular vesicles (EVs), of the ‘brown stomach worm’ *Teladorsagia circumcincta*, one of the major agents causing parasite gastroenteritis in temperate areas worldwide; (ii) conduct bioinformatic analyses to identify and characterise antimicrobial peptides (AMPs) with putative antimicrobial activity; and (iii) assess the bactericidal and/or bacteriostatic properties of *T. circumcincta* EVs, and whole and EV-depleted ESPs, using bacterial growth inhibition assays.

**Methods:**

Size-exclusion chromatography was applied to the isolation of EVs from whole *T. circumcincta* ESPs, followed by EV characterisation via nanoparticle tracking analysis and transmission electron microscopy. Proteomic analysis of EVs and EV-depleted ESPs was conducted using liquid chromatography-tandem mass spectrometry, and prediction of putative AMPs was performed using available online tools. The antimicrobial activities of *T. circumcincta* EVs and of whole and EV-depleted ESPs against *Escherichia coli* were evaluated using bacterial growth inhibition assays.

**Results:**

Several molecules with putative antimicrobial activity were identified in both EVs and EV-depleted ESPs from adult *T. circumcincta*. Whilst exposure of *E. coli* to whole ESPs resulted in a significant reduction of colony-forming units over 3 h, bacterial growth was not reduced following exposure to worm EVs or EV-depleted ESPs.

**Conclusions:**

Our data points towards a bactericidal and/or bacteriostatic function of *T. circumcincta* ESPs, likely mediated by molecules with antimicrobial activity.

**Graphical Abstract:**

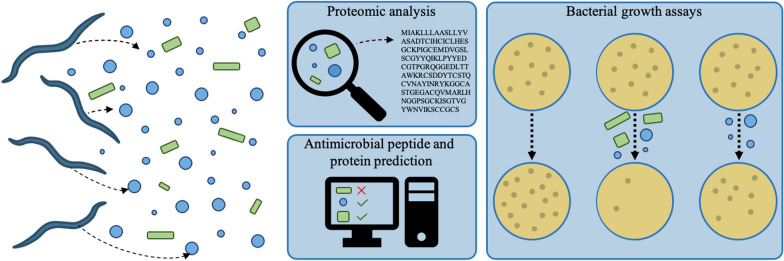

**Supplementary Information:**

The online version contains supplementary material available at 10.1186/s13071-022-05443-z.

## Background

The complex network of interactions between gastrointestinal (GI) helminths of ruminant livestock and their vertebrate hosts has been the subject of intense research over the past several decades, owing to the increasing demand for novel and sustainable methods of parasite control in the face of widespread anthelmintic resistance [1–5]. Of these interactions, the crosstalk between GI parasitic worms and the resident gut microbiota is receiving increasing attention, primarily because of the key role(s) that the latter exerts in nutrient processing and absorption, protection against pathogens and, in ruminants in particular, conversion of digesta into meat and dairy [6–9]. Indeed, several studies to date [10–13] have shown that infections by GI helminths are associated with profound modifications of the composition of the ruminant gut microbiome and relative abundance of individual microbial species, with likely repercussions on the pathophysiology of helminth disease, efficiency of food conversion and overall host health and welfare. Nevertheless, data generated in these studies are highly variable and, in some instances, contradictory, likely because of several intrinsic and environmental factors that shape the composition of the vertebrate gut microbiota [13]. For instance, we first reported that experimental infection of 6- to–9 months-old lambs with the ‘brown stomach worm’, *Teladorsagia circumcincta*, resulted in significantly altered faecal microbial profiles when compared with uninfected control animals; in particular, bacteria belonging to the genera *Porphyromonas*, *Sutterella*,* Prevotella* and *Bacteroidales* RF16 group were expanded in faecal samples from worm-infected lambs, and accompanied by progressively decreasing bacterial beta diversity. These observations had led us to hypothesise that a rise in gastric pH, which often follows establishment of *T. circumcincta*, might facilitate the survival of selected ruminal bacteria transiting through the abomasum of worm-colonised animals [12]. In an attempt to assess the reproducibility of these findings, we conducted a second study under similar experimental protocols over the following grazing season [13]; data from the latter study were not consistent with those from Cortés et al*.* [12]. For example, we observed a significant reduction of *Akkermansia* in faeces of *T. circumcincta*-infected lambs [13] that had not been detected previously [12], whilst bacterial beta diversity was increased in faeces from these animals, albeit with no consistent trend between consecutive time points [13]. These discrepancies represent a significant challenge to attempts to derive meaningful information from in vivo helminth-microbiome relationship studies, and to assess the impact of such interactions on animal production (reviewed by [14]). Improving current understanding of the mechanisms underpinning the interactions between GI helminths, host and gut microbiota is nonetheless pivotal, as it will lead to translational studies aimed to exploit the latter as means of controlling GI worms of livestock.

Several hypotheses have been raised on the mechanisms regulating the microbiota-modulating properties of GI helminths (reviewed by [15]). These include both indirect and direct interactions, via parasite-elicited local and systemic immune responses, and parasite excreted-secreted products (ESPs) with antimicrobial activity (reviewed by [15]). For instance, ESPs from the murine nematode *Heligmosomoides polygyrus* exert antimicrobial activity against several Gram-negative and -positive bacteria, including *Escherichia coli*, *Salmonella enterica* serovar Typhimurium, *Enterococcus faecium* and *Staphylococcus aureus* [16]; similarly, ESPs from the swine roundworm *Ascaris suum* contain several antimicrobial peptides and proteins (AMPs) and induce dose-dependent inhibition of biofilm formation by biofilm-forming *E. coli* K-12 strains [17]. For the latter, in particular, a recent proteomic analysis localised putative AMPs to the ESP fraction containing secreted extracellular vesicles (EVs) [18]. Helminth EVs are defined as lipid membrane-enclosed packages, rich in nucleic acids, peptide and protein cargo [19], with several functions in host-parasite interactions, including helminth migration through host tissues, nutrition (via the activity of protein-degrading enzymes) and modulation of host immune responses [19, 20]. Crucially, in a recent study, exposure of rumen fluids to EVs derived from the rumen fluke, *Calicophoron daubneyi*, resulted in increased bacterial diversity, which led the authors to speculate that *C. daubneyi* EVs might exert bactericidal and/or bacteriostatic activities that be (at least partially) responsible for the changes in gut microbial profiles observed over the course of parasite infection and establishment [21].

In spite of emerging evidence of the role(s) of helminth ESPs and EVs in parasite-microbiota crosstalk, little is known of the putative antimicrobial properties of molecules contained in ESPs of livestock helminths, including those enclosed in vesicles [21–23]. Whilst previous investigations have comprehensively elucidated the proteomic profiles of ESPs derived from third- and fourth-stage larvae of *T. circumcincta* (L3s and L4s, respectively) [24], only a single study has undertaken a preliminary proteomic analysis of EV-enriched ESPs (obtained by ultracentrifugation) from L4s of this parasite [25]. This study led to the identification of several molecules with putative functions in host-helminth communication, including proteases, antioxidants, ATPases and a saposin B. The latter is a member of a lysosome-associated family of non-enzymatic glycoproteins with known roles in lipid-antigen presentation to CD1-restricted T cells, processing of apoptotic bodies for antigen delivery and antimicrobial defence [26]. The detection of molecules with putative antibacterial properties in the EV-enriched fraction of *T. circumcincta* ESPs supports a likely role of parasite secretions in helminth-microbiota crosstalk, and provides a solid rationale for experiments aimed to assess the antibacterial activities of ESPs and EVs in vitro (cf. [23]). In the present study, we (i) undertook an in-depth proteomic characterisation of adult *T. circumcincta* EVs isolated via size-exclusion chromatography (SEC) and of EV-depleted ESPs; (ii) performed targeted bioinformatics analyses of EV-depleted ESP- and EV-derived amino acid sequence data in order to identify molecules with putative antimicrobial functions; and (iii) assessed the bactericidal and/or bacteriostatic properties of adult *T. circumcincta* whole and EV-depleted ESPs, and SEC-purified EVs in in vitro bacterial growth viability assays.

## Methods

### Parasite material and production of ESPs

Helminth-free Texel cross lambs (< 6 months old) were challenged with 20,000 infective *T. circumcincta* L3s per lamb (isolate MTci-2_CVL). At 28 days post-infection, the animals were euthanised, and lumen-dwelling *T. circumcincta* adult male and female parasites were collected as previously described [27]. Briefly, lumen-dwelling harvested parasites were washed three times in phosphate-buffered saline (PBS) before culturing in RPMI 1640 medium (Invitrogen™, Thermo Fisher Scientific, Waltham, MA, USA) containing 20 mM HEPES pH 7.5, 1% (*w*/*v*) d-glucose, 2 mM l-glutamine, 1000 U/ml penicillin, 1000 μg/ml streptomycin, 200 μg/ml gentamycin and 25 μg/ml amphotericin B, at 37 °C in 5% CO_2_. Culture supernatants were harvested every 24 h and replaced with fresh media; supernatants from collections performed at 96 and 120 h were used in this study. At each time point, parasite viability was confirmed on the basis of structural integrity and motility. The culture supernatants were clarified by centrifugation at 300 *g* for 10 min at 4 °C and filtered through a 0.2-μm Minisart® syringe filter (Sartorius AG, Göttingen, Germany) and stored at − 70 °C. For processing of ESPs, culture supernatants were defrosted and centrifuged at 4 °C at 300 *g* for 10 min and subsequently at 700 *g* for 30 min. The supernatant, containing *T. circumcincta* ESPs, was transferred into a separate Falcon tube and concentrated using 10-kDa MWCO Amicon Ultra-15 Centrifugal Filter Units (MilliporeSigma, Burlington, MA, USA), following the manufacturer’s guidelines. In order to ensure complete removal of antibiotic residues derived from the culture media, concentrated *T. circumcincta* ESPs were buffer exchanged 5 times into filtered PBS (0.2 μm; Life Sciences, Thermo Fisher Scientific). Protein concentration was determined using the Pierce™ BCA protein assay (Thermo Fisher Scientific) with bovine serum albumin standards, and aliquots of ESPs were stored at − 70 °C prior to use.

### Isolation and purification of EVs from parasite ESPs

Isolation and purification of EVs from ESPs obtained from adult male and female *T. circumcincta* was carried out as previously described [28]. Approximately 150 μl of concentrated *T. circumcincta* ESPs was filtered through qEVsingle size exclusion chromatography (SEC) columns (IZON Science, Christchurch, New Zealand), using the manufacturer’s optimised protocol. Briefly, each SEC unit was flushed with the equivalent of three full column volumes of filtered PBS, thus ensuring complete removal of the column storage buffer. The concentrated *T. circumcincta* ESPs were added to the column, and the first 1 ml of flowthrough was discarded. The following 0.6 ml of flowthrough, containing EVs, was collected and stored at − 80 °C. Finally, the remaining 3 ml of flowthrough, containing EV-depleted ESPs, was collected and stored at − 80 °C until further use.

### Transmission electron microscopy and nanoparticle tracking analysis of *T. circumcincta* EVs

For transmission electron microscopy (TEM), 10 μl of each *T. circumcincta* whole ESPs and EV fractions was adsorbed onto glow-discharged 400 mesh copper/carbon film grids (EM Resolutions Ltd., Sheffield, UK) for approximately 1 min. TEM grids were subsequently washed with two drops of deionised water to remove buffer salts and stained with 2% (w/v) aqueous uranyl acetate dye for approximatly 30 s. The latter was drained using filter paper, and grids were allowed to air dry. Adult *T. circumcincta* EVs were visualised using a Tecnai G20 transmission electron microscope (FEI/Thermo Fisher Scientific) at an accelerating voltage of 200 keV using a 20-μm objective aperture to improve contrast. Images were acquired using an AMETEK CCD camera (AMETEK Inc., Berwyn, PA, USA). In addition to TEM visualisation, adult *T. circumcincta* EV size and concentration were assessed using a NanoSight NS300 instrument for nanoparticle tracking analysis (NTA) (Malvern Panalytical, Malvern, UK). Briefly, EVs were diluted (1 in 10) in filtered PBS to achieve a suitable volume for loading onto the NanoSight instrument with a syringe. Although lower-than-optimal particles per frame were recorded (i.e. < 30), no concentration warnings were detected. For measurement of concentration, three 60 s videos were captured under the following conditions: temperature = 25 °C; syringe speed = 100 AU; camera level = 15; hardware: camera type = sCMOS; laser type = Blue488. Videos were subsequently analysed using the in-built NanoSight Software NTA Build 3.2 with a detection threshold of 5. Average particle size and concentration were measured for each video using the NanoSight software. The final particle size was reported as the mean of the three measurements, in nanometres (± standard error of the mean [SEM]), whilst the final particle concentration was reported as the mean of the three measurements in particles/ml (± SEM).

### Proteomic analysis of *T. circumcincta* EVs and EV-depleted ESPs

A 10-µg aliquot of each EV-depleted ESP and EV fraction was run on 12.5% v/v one-dimensional (1D) sodium dodecyl sulphate polyacrylamide gel electrophoresis (SDS-PAGE) gels using the Protean® II xi 2-D Cell (Bio-Rad Laboratories Inc., Hercules, CA, USA). Samples were mixed with SDS loading buffer (0.2 M Tris–HCl, 8% SDS, 40% glycerol, 0.02% bronophenol blue, 50 mM DTT, pH 6.8), heated at 95 °C for 5 min and loaded. 1D gels were run at 70 V for 30 min followed by 150 V for 1 h. Gels were stained with 10% v/v acetic acid and 40% v/v ethanol for 1 h followed by overnight staining with Colloidal Coomassie blue (Phastgel Blue R, Amersham Biosciences, Amersham, UK). Subsequently, gels were de-stained in 1% v/v acetic acid and imaged using a GS-800 calibrated densitometer (Bio-Rad Laboratories). 1D gel lanes containing visible protein bands were manually excised into 10 equal sections. Gel excises were washed and de-stained in a solution of 50% v/v 50 mM ammonium bicarbonate and 50% v/v acetonitrile (ACN) for 15 min at 37 °C, and subsequently dehydrated with the addition of 100% ACN followed by incubation at 50 °C. The dehydrated gel excises were digested overnight at 37 °C using proteomic grade trypsin (Sigma-Aldrich, St. Louis, MO, USA) diluted to 10 ng/µl in 50 mM ammonium bicarbonate. Peptides were subsequently extracted by shaking gels at room temperature in 50 µl milli-Q water for 10 min followed by the addition of 50 µl of 50% v/v ACN and 5% v/v formic acid in milli-Q water and shaking for 60 min. Eluants were dried by vacuum centrifugation and re-suspended in 20 µl 0.1% v/v formic acid immediately prior to liquid chromatography–tandem mass spectrometry analysis (LC–MS/MS).

LC–MS/MS was conducted using a Orbitrap Fusion™ Tribrid™ mass spectrometer (Thermo Fisher Scientific), with EASY-Spray™ source, coupled to an UltiMate™ 3000 RSLCnano system (Thermo Fisher Scientific). Liquid chromatography was conducted with a Thermo Fisher Scientific EASY-Spray™ ES905 HPLC column C18 (75 μm × 750 mm, with 2-μm particle size). The mobile phases for gradient elution were ultrapure water (18.2 Ω) with 0.1% formic acid as eluant A, and 80% acetonitrile with 0.1% formic acid as eluant B. Liquid chromatography was performed with a flow rate of 200 nl/min, starting with 3% eluant B, then at 0.5 min until 50 min rising to 30%, 30–40% over 5 min, then rising to 95% eluant B over a further 15 min and held for 10 min before equilibration at 3% for 30 min. Ions were generated with a source voltage of 1800 V in positive mode, with an ion transfer temperature of 275 °C. Standard peptide analysis parameters were used; parent ions were detected in profile mode in the 375–1500 *m*/*z* range in the orbitrap at a resolution of 120,000 and a maximum injection time of 50 ms in positive mode. MS^2^ data were collected in data dependent mode including charge states of 2–7. Dynamic exclusion of masses was conducted for 20 s after initial selection for MS^2^. Ions were formed by fragmentation by collision-induced dissociation with a collision energy of 35%. Resulting ions were detected in the ion trap in centroid mode.

Peak lists were produced and exported as Mascot Generic Files (MGF) using MSConvert (version 3.0) [29]. Data analysis was performed using the Mass Hunter Qualitative Analysis software (vB.06; Agilent Technologies Inc., Santa Clara, CA, USA) as previously described [30]. Briefly, the data were queried against *T. circumcincta* gene sequences, accessed via WormBase ParaSite (http://parasite.wormbase.org/, BioProject PRJNA72569, version WBPS16), using the MASCOT daemon (v2.4.1; Matrix Science, London, UK) MS/MS ions search. The following search parameters were used: enzyme to trypsin with one missed cleavage allowed; fixed modifications to carbamidomethylation with variable modifications set for oxidation of methionine; and fixed monoisotopic masses with unrestricted protein masses with peptide and fragment mass tolerances at ± 1.2 Da and ± 0.6 Da, respectively. Protein identifications were reported at a false discovery rate (FDR) of 1%. The complete list of peptides identified in EV and EV-depleted fractions of *T. circumcincta* ESPs is provided in Additional file 1: Table S1. All *T. circumcincta* EV and EV-depleted ESP protein sequences were further analysed using the BLAST2GO software (https://www.blast2go.com/); the tBLASTn and BLASTp functions were applied to the identification of sequences within the nucleotide and protein collection databases of NCBI with significant similarity to EV and EV-depleted ESP protein sequences, using the following parameters: no taxonomy filter; *E*-value cut-off: 1.0E^−5^; BLAST description annotator: on; Word size: 3; Low complexity filter: on; HSP length cut-off: 33; HSP-hit coverage: 0. EV and EV-depleted ESP amino acid sequences were assigned InterProScan identifiers (IDs) and Gene Ontology (GO) terms, according to the categories ‘Biological Process’ (BP), ‘Cellular Component’ (CC), and ‘Molecular Function’ (MF), using BLAST2GO annotation results. GO mapping parameters were kept as default. Sequences were manually screened for contaminants against the GPM cRAP contaminant database (https://www.thegpm.org/crap/); no contaminant sequences were detected.

### Antimicrobial peptide and protein prediction

All *T. circumcincta* protein sequences identified in the EV and EV-depleted ESP fractions were subjected to prediction of antimicrobial activity via available online software. First, all sequences were individually uploaded onto the Collection of Anti-Microbial Peptides (CAMP_R3_) database website (http://www.camp.bicnirrh.res.in/) [31]. The BLASTp tool in CAMP_R3_ was applied to the identification of significant similarities between *T. circumcincta* EV and EV-depleted ESP amino acid sequences and sequences available in the database using the following parameters: matrix = BLOSUM62; alignment = ungapped; *E*-value threshold = 1E^−5^. In cases of multiple significant alignments for the same *T. circumcincta* sequence, only the top scoring hit was retained. All *T. circumcincta* EV and EV-depleted ESP amino acid sequences were individually uploaded onto the AMP prediction tool, ampir (https://ampir.marine-omics.net/). This tool assigns a probability score (between 0 and 1, where 1 indicates the highest probability of antimicrobial activity) to each sequence based on physico-chemical properties of the respective amino acid patterns using a supervised statistical machine learning approach [32]. ampir supports AMP prediction using two vector machine classification models, i.e. “precursor” (best suited for analysis of full-length proteins), and “mature” (for sequences representing the final AMP sequence after post-translational processing, e.g. removal of N-terminal signal and pro-peptide sequences) [32]. ampir probability scores were recorded for each *T. circumcincta* EV and EV-depleted ESP amino acid sequence using both classification models. Sequences were subsequently classified as either ‘mature’ or ‘precursor’ using parameters suggested by ampir, and the corresponding scores were taken into account for AMP prediction. Sequences returning ampir scores > 0.7 were retained as ‘sequences of interest’ (see below).

A further AMP prediction tool, MultiPep (https://github.com/scheelelab/MultiPep), was applied to the identification of *T. circumcincta* EV and EV-depleted ESP sequences with putative antimicrobial properties. MultiPep assigns each query sequence to one of 20 peptide bioactivity classes, based on intrinsic amino acid patterns [33]. Then, within each bioactivity class, individual sequences are assigned a probability score of between 0 and 1, where 1 indicates the highest probability of a given query sequence correctly identified as belonging to the corresponding bioactivity class. Since MultiPep limits analyses to sequences ranging from two to 200 amino acids in length, EV and EV-depleted ESP sequences that did not meet the length requirements were not included in the analysis. The ‘MultiPep_predict.py’ python script was executed as instructed by the software developer. For each MultiPep-compatible *T. circumcincta* sequence, antimicrobial and antibacterial probability scores were recorded; sequences that returned scores > 0.4 for either antimicrobial and/or antibacterial probability were retained as ‘sequences of interest’ (see below). Finally, each EV and EV-depleted ESP amino acid sequence was analysed using the Antimicrobial Sequence Scanning System (AMPA; https://tcoffee.crg.eu/apps/ampa/guide.html) using default settings. The AMPA algorithm identifies regions within a sequence with likely antimicrobial activity that are subsequently assigned an ‘antimicrobial propensity value’ (i.e. the probability of such a region to occur in an antimicrobial molecule) and a ‘misclassification probability value’ (i.e. the probability of such a region to occur by chance in a non-antimicrobial protein) [34].

*Teladorsagia circumcincta* EV and EV-depleted ESP amino acid sequences with the highest antimicrobial probability scores according to 1 or more AMP prediction tools or those that were retained as ‘sequences of interest’ according to at least 2 AMP prediction tools were compiled in a list of putative antimicrobial proteins and are discussed below (cf. section Proteomic analyses of *T. circumcincta* EVs and EV-depleted ESPs, and AMP predictions).

### Bacterial growth assays

To assess the bactericidal/bacteriostatic properties of adult *T. circumcincta* EVs and EV-depleted ESPs, BL21 *E. coli* bacteria were transformed with the Bioware pXEN13 plasmid containing the luxCDABE operon by electroporation, as previously described [35], and transformants were selected on a Luria-Bertani (LB) agar plate containing 100 μg/ml ampicillin. Following transformation, a single bacterial colony was selected and used to inoculate 10 ml of LB broth containing 100 μg/ml ampicillin, and cultured for 6 h at 37 °C with shaking at 250 rpm. The OD_600_ for the culture was measured after 6 h and initially diluted to OD_600_ = 0.15, and subsequently to 1/5000. The 1/5000 bacteria dilution was used in bacterial colony counting experiments. Bacterial growth assays were run in triplicate for each condition, using a 96-well plate. Briefly, 10 μl of the 1/5000 bacteria culture and 90 μl of LB broth were added to each replicate. Given the low concentrations of whole ESPs, EV and EV-depleted ESPs, the maximum available volume of *T. circumcincta* material was added to the wells, which resulted in slightly different concentrations of whole ESPs and EV-depleted ESPs added to the assay, i.e. 28 μg/ml for *T. circumcincta* ESPs, 3.65 × 10^8^ particles/ml for *T. circumcincta* EVs and 21 μg/ml of *T. circumcincta* EV-depleted ESPs. PBS replaced parasite material in negative control wells. In order to assess the impact of low initial concentrations of *T. circumcincta* material on the outcome of bacterial growth assays, we performed an additional proof-of-concept, follow-up experiment utilising SEC-purified EVs and EV-depleted ESPs from the liver fluke, *Fasciola hepatica*. Our choice was based on several considerations. First, due to the considerably larger size of *F. hepatica* adult worms compared with *T. circumcincta* (i.e. 30 vs 12 mm in length, respectively), as well as the presence of a tegumental surface (a cuticle in *T. circumcincta*) that is known to shed ESPs [19], larger quantities of ESP material could readily be obtained from the former. Critically, SEC-purified EVs from *C. daubneyi*, which is phylogenetically closely related to *F. hepatica*, have recently been demonstrated to exert clear antimicrobial activity [21]. Fasciolosis, like teladorsagiosis, has been previously shown to be associated with alterations of the ruminant gut microbiota [36]. Finally, current research conducted in the Morphew laboratory (Aberystwyth, UK; unpublished) has provided evidence that *F. hepatica* ESPs and EVs exert antimicrobial activity in vitro. Experimental protocols identical to those outlined above for *T. circumcincta* were applied to the purification of *F. hepatica* ESPs from starting material available in the RM laboratory (Aberystwyth, UK); *F. hepatica* EVs were isolated using SEC and diluted (1 in 100) using filtered PBS, characterised and quantified using NTA (Additional file 2: Figure S1). Two concentrations for each *F. hepatica* whole ESPs, SEC-purified EVs and EV-depleted ESPs were used in the follow-up experiment, i.e. a ‘high’ and a ‘low’ concentration, with the latter equivalent to available *T. circumcincta* corresponding materials. In particular, ‘high’ and ‘low’ concentrations amounted to 74.85 and 28 μg/ml for *F. hepatica* whole ESPs; 1.63 × 10^10^ and 3.65 × 10^8^ particles/ml for SEC-purified EVs; and 61 and 21 μg/ml for EV-depleted ESPs, respectively. Volumes were standardised to 200 μl using filtered PBS. Bacteria were subsequently incubated at 37 °C with shaking at 250 rpm for 3 h.

The Miles and Misra method was used for colony counting, as previously described [35]. Briefly, serial dilutions of the 3 h bacterial cultures were spotted onto ampicillin-containing agar plates, allowed to dry and then incubated at 37 °C for 18 h. For each of the three replicates, colony counts were performed from the highest bacterial dilution that yielded distinct colonies. The following equation was applied to calculate the number of colony-forming units (CFU)/ml in each replicate: CFU/ml = (number of colonies × 50 × dilution factor)/20. The CFU/ml values for each well condition were analysed using GraphPad Prism version 5.01 software (GraphPad Software, San Diego, CA, USA), and compared using a one-way analysis of variance with Tukey’s multiple comparison test to identify significant differences between the conditions. A *P* ≤ 0.05 reduction in CFU/ml values when compared to the negative control was considered to be statistically significant.

## Results

### Confirmation and visualisation of EVs in* T. circumcincta* ESPs

**Fig. 1:**
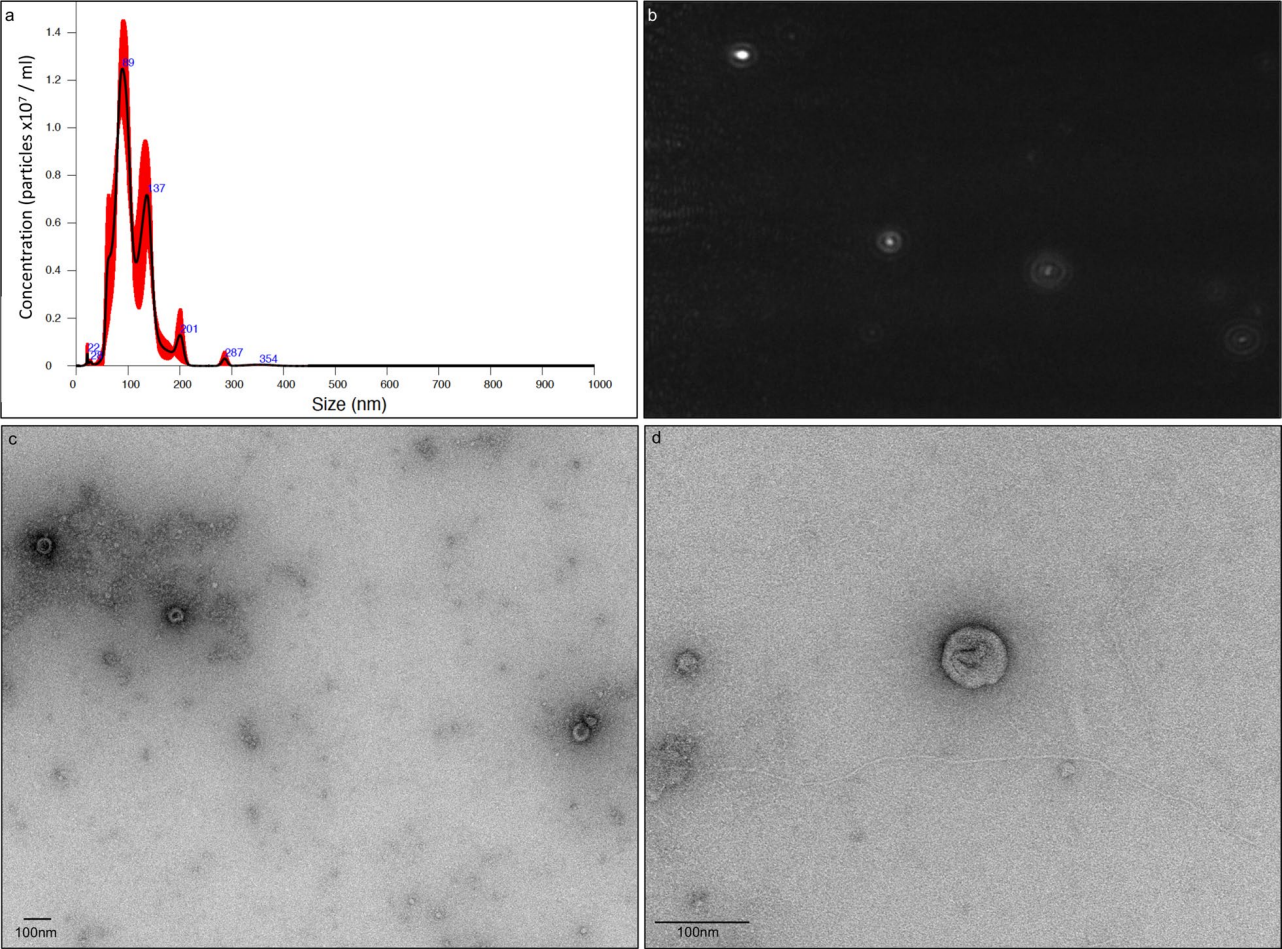
Visual characterisation of *Teladorsagia circumcincta* extracellular vesicles (EVs) using nanoparticle tracking analysis (NTA) and transmission electron microscopy (TEM). **a** Size distribution of size-exclusion chromatography (SEC)-purified EVs from excretory-secretory products (ESPs) of adult *T. circumcincta*, according to NTA performed on a NS300 instrument. **b** Representative image of SEC-purified EVs from adult *T. circumcincta* captured by the NanoSight NS300 instrument (error bars indicate standard error of the mean). **c** TEM images of EVs from whole ESPs of adult *T. circumcincta* and **d** SEC-purified EVs. Scale bars are provided at the bottom left corner of **c** and **d**

Protein concentrations of 577 and 43 μg/ml were achieved for whole *T. circumcincta* ESPs and EV-depleted ESPs, respectively. The concentration and morphology of *T. circumcincta* EVs following SEC purification were assessed using NTA and TEM (Fig. 1a–d). By NTA, average (± standard error [SE]) particle concentration of diluted (1 in 10) *T. circumcincta* EVs was 7.29 × 10^7^ (± 4.92 × 10^6^) particles/ml, leading to a final concentration of 7.29 × 10^8^ particles/ml. Average particle diameter was 108.7 (± 3.3) nm, with an average of 10% particles sized at ≤ 70.6 (± 5) nm in diameter, 50% at ≤ 98.9 (± 2.3) nm and 90% at ≤ 158.0 (± 12.5) nm, respectively (Fig. 1a).

The presence of EVs in *T. circumcincta* whole ESPs and EV fractions was analysed by TEM (Fig. 1c and d, respectively). Within whole ESPs, EVs ranged between 50 and 100 nm in diameter, consistent with the size of exosomes. The exosome-like particles typically appeared as ‘cup shaped’, likely due to dehydration during sample preparation [37]. Exosome-like particles were also visible within the EV fraction of *T. circumcincta* ESPs, albeit with fewer particles readily detected, likely due to sample dilution during the SEC purification step.

### Proteomic analyses of* T. circumcincta* EVs and EV-depleted ESPs, and AMP predictions

Proteomic analyses of *T. circumcincta* EVs and EV-depleted ESPs yielded 55 and 423 amino acid sequences, respectively. The complete lists of sequences identified, together with top tBLASTn/BLASTp hit (NCBI nt/nr database), InterProScan analysis, GO annotation and results of AMP prediction analyses (based on CAMP_R3_ BLASTp, ampir and MultiPep) are provided in Additional file 3: Table S2 and Additional file 4: Table S3, respectively. Results from AMPA analyses are available from Additional file 5: Table S4. A total of 23 sequences were shared between the two fractions, while the remainders were unique to each (cf. Additional file 3: Table S2 and Additional file 4: Table S3).

Of the amino acid sequences identified from *T. circumcincta* EVs, five yielded high antimicrobial activity prediction scores according to one or more AMP prediction software, i.e. TELCIR_16939, TELCIR_03997, TELCIR_02518, TELCIR_02918 and TELCIR_14644 (Table 1). Of these, TELCIR_16939 returned the highest ampir score (i.e. 0.73), with tBLASTn analysis revealing significant similarity to an '*Ancylostoma*-secreted protein-like protein’ from the bovine stomach worm *Ostertagia ostertagi* (*E*-value: 1.57E^−77^; cf. Table 1). A 14-amino acid-long putative antimicrobial region spanning residues 152–165 was identified by AMPA (Additional file 5: Table S4). InterProScan annotation assigned TELCIR_16939 to the cysteine-rich secretory proteins, antigen 5, and pathogenesis-related 1 proteins (CAP) superfamily (cf. Table 1). Whilst ampir nor MultiPep returned significantly high scores for TELCIR_03997 and TELCIR_02518, CAMP_R3_ BLASTp revealed significant similarities between these sequences and those of a beta-defensin (3.00E^−8^) and colicin-E3 (1.00E^−6^), respectively. tBLASTn annotation identified TELCIR_03997 as a homologue of the citron homology (CNH) domain protein from the human hookworm, *Necator americanus* (*E*-value: 0; cf. Table 1). InterProScan annotation identified a AGC-kinase domain, alongside a Cdc42/Rac interactive binding (CRIB) domain and protein kinase domain, whilst GO annotation returned the terms ‘protein phosphorylation’ (BP) and ‘ATP binding’ and ‘protein serine/threonine kinase activity’ (MF) (cf. Additional file 3: Table S2). TELCIR_02518 was identified as a homologue of a hypothetical protein from the ‘barber’s pole worm’, *Haemonchus contortus* (*E*-value: 1.50E^−10^) (Table 1). TELCIR_02918 displayed significant similarity to a region of the *Parastrongyloides trichosuri* genome assembly (cf. Table 1). Whilst this sequence did not match any of the available sequences within the CAMP_R3_ database, nor returned a significantly high ampir score, it was identified as a likely antibacterial molecule by MultiPep (cf. Table 1) and was predicted to include a 21-amino acid-long antimicrobial region by AMPA (Additional file 5: Table S4). TELCIR_02918 belongs to the ABC transporter A protein family and contains an ABC transporter-like, ATP-binding domain (cf. Table 1). GO terms assigned to this sequence include ‘transmembrane transport’ (BP) and ‘ABC-type transporter activity’ and ATP binding (MF) (cf. Additional file 3: Table S2). Similarly to TELCIR_02918, TELCIR_14644 was predicted to exert antibacterial activity by MultiPep. This sequence displays significant similarity to a *Strongylocentrotus purpuratus* histone H4-like protein (*E*-value: 1.61E^−36^; cf. Table 1). GO annotation returned the terms ‘DNA binding’ and ‘protein heterodimerisation activity’ (MF) (cf. Additional file 3: Table S2).

**Table 1:** Adult *Teladorsagia circumcincta* extracellular vesicles (EVs) and extracellular vesicle-depleted excretory-secretory products (EV-depleted ESPs) are predicted to contain molecules with antimicrobial activity

Protein Identifier	Top tBLASTn hit	Accession number	CAMP_R3_ BLAST hit	ampir score	MultiPep antimicrobial score	MultiPep antibacterial score
*EV sequences*						
TELCIR_16939	*Ostertagia ostertagi* mRNA for *ancylostoma*-secreted protein-like protein (*aasp2* gene)	AJ515523		0.731774777	0.002533278	0.00026482
TELCIR_03997	*Necator americanus* CNH domain protein mRNA	XM_013439788	Beta-defensin	0.036062437		
TELCIR_02518	*Haemonchus contortus* strain NZ_Hco_NP chromosome 4	CP035804	Colicin-E3	0.034655877	0.006489962	0.00038019
TELCIR_02918	*Parastrongyloides trichosuri* genome assembly *P_trichosuri*_KNP, scaffold PTRK_scaffold0000014	LM523171		0.001274926	0.45438498	0.6185435
TELCIR_14644	PREDICTED: *Strongylocentrotus purpuratus* histone H4-like (LOC763704), mRNA	XM_030973888		0.000623	0.30540183	0.4312727
*EV-depleted ESP sequences*						
TELCIR_10665	*Haemonchus contortus* strain NZ_Hco_NP chromosome 5	CP035803		0.979139815	0.06761581	0.015811814
TELCIR_11549	*Haemonchus contortus,* ISE/inbred ISE, WGS project CAVP01000000 data, chromosome: 4	LS997565		0.964016656	0.257768	0.66219217
TELCIR_24131	*Haemonchus contortus* strain NZ_Hco_NP chromosome 4	CP035804		0.956493679	0.7135719	0.6023187
TELCIR_16412	*Necator americanus* destabilase mRNA	XM_013442357	Lysozyme 3	0.922788325	0.4397563	0.04875455
TELCIR_17750	PREDICTED: *Biomphalaria glabrata* myophilin-like (LOC106058506), partial mRNA	XM_013215952		0.907709915	0.41452104	0.56613505
TELCIR_20259	*Necator americanus* hypothetical protein partial mRNA	XM_013449087		0.871642795	0.7324507	0.35694736
TELCIR_13940	*Ostertagia ostertagi* mRNA for C-type single domain activation associated secreted protein ASP3 precursor (*asp3*)	AM747038		0.807793852	0.47152907	0.18022908
TELCIR_24691	*Schistosoma mansoni* ubiquitin mRNA, partial cds	AY485340	CgUbiquitin	0.710008917	0.5021459	0.6605984

Proteins were identified from size-exclusion chromatography (SEC)-purified extracellular vesicles (EVs) from adult *T. circumcincta* excretory-secretory products (ESPs), as well as from EV-depleted ESPs; putative antimicrobial activity was determined on the basis of comparative analyses with sequence data available from the Collection of Anti-Microbial Peptides (CAMP_R3_) database, as well as according to antimicrobial peptide (AMP) prediction algorithms (ampir and MultiPep). For each sequence, the top homologues in the National Center for Biotechnology Information (NCBI; nucleotide collection database), with corresponding accession number, and CAMP_R3_ databases are provided as well as ampir and MultiPep prediction scores

Proteomic analyses of *T. circumcincta* EV-depleted ESPs led to the identification of eight molecules with putative antimicrobial activity, i.e. TELCIR_24691, TELCIR_16412, TELCIR_10665, TELCIR_11549, TELCIR_20259, TELCIR_13940, TELCIR_17750 and TELCIR_24131 (Table 1). TELCIR_24691 was predicted to exert antimicrobial activity by all of the antimicrobial prediction tools, with an ampir score of 0.71, and antimicrobial and antibacterial MultiPep scores of 0.50 and 0.66, respectively. TELCIR_24691 was predicted to include two regions, spanning 14 and 19 amino acids respectively, with putative antimicrobial activity (Additional file 5: Table S4). In addition, this sequence shared significant similarity with a ubiquitin from the blood fluke *Schistosoma mansoni* (*E*-value: 3.89E^−50^), as well as to a CgUbiquitin from *Crassostrea gigas* in the CAMP_R3_ database (*E*-value: 9.00E^−47^) (cf. Table 1). InterProScan analysis led to the detection of both a ubiquitin-like domain and a ubiquitin conserved site that were assigned the GO term ‘protein binding’ (MF) (cf. Additional file 4: Table S3). TELCIR_16412, a homologue of a *N. americanus* destabilase (*E*-value: 4.96E^−73^), was predicted to exert antimicrobial activity by both ampir and MultiPep, a finding consistent with the significant homology detected between this sequence and a lysozyme 3 protein sequence from *Crassostrea virginica* available in the CAMP_R3_ database (*E*-value: 4.00E^−39^), as well as with the detection of a ‘invertebrate-type lysozyme’ protein domain by InterProScan. This sequence was assigned the GO term ‘lysozyme activity’ (MF) (cf. Additional file 4: Table S3).

TELCIR_10665 was the highest ampir-scoring sequence (ampir score: 0.98) and shared significant similarity to a region of *H. contortus* chromosome 5 (*E*-value: 1.73E^−9^). Aside from the identification of a signal peptide and a non-cytoplasmic domain, no further annotation could be assigned (cf. Additional file 4: Table S3). TELCIR_11549, a sequence sharing significant similarity to a region of the *H. contortus* chromosome 4 (*E*-value 1.47E^−18^), returned an ampir score of 0.96 and a MultiPep antibacterial score of 0.66, and was predicted to include a region with antimicrobial function by AMPA (cf. Table 1; Additional file 5: Table S4). TELCIR_20259 was predicted to exert antimicrobial activity by both ampir and MultiPep (scores: 0.87 and 0.73, respectively); however, no further annotation could be assigned.

TELCIR_13940 and TELCIR_17750 returned high antimicrobial activity probability scores according to both ampir and MultiPep (i.e. 0.81 and 0.47, and 0.91 and 0.41, respectively). The former shared significant similarity to an *O. ostertagi* messenger RNA (mRNA) C-type single-domain activation-associated secreted protein (ASP3) precursor (*E*-value: 1.70E^−36^) (cf. Table 1), while the latter yielded significant similarity to a myophilin-like protein from the snail *Biomphalaria glabrata* (*E*-value: 2.19E^−40^). InterProScan analysis of TELCIR_17750 identified a calponin homology domain spanning the majority of the sequence (cf. Additional file 4: Table S3). Finally, TELCIR_24131 returned high antimicrobial scores for both ampir and MultiPep (i.e. 0.95 and 0.71, respectively). This sequence, with significant similarity to a region within the *H. contortus* chromosome 4 (*E*-value: 2.26E^−15^), was identified as belonging to the transthyretin-like family (cf. Table 1).

### Antibacterial activity of *T. circumcincta* EVs, and whole and EV-depleted ESPs

**Fig. 2:**
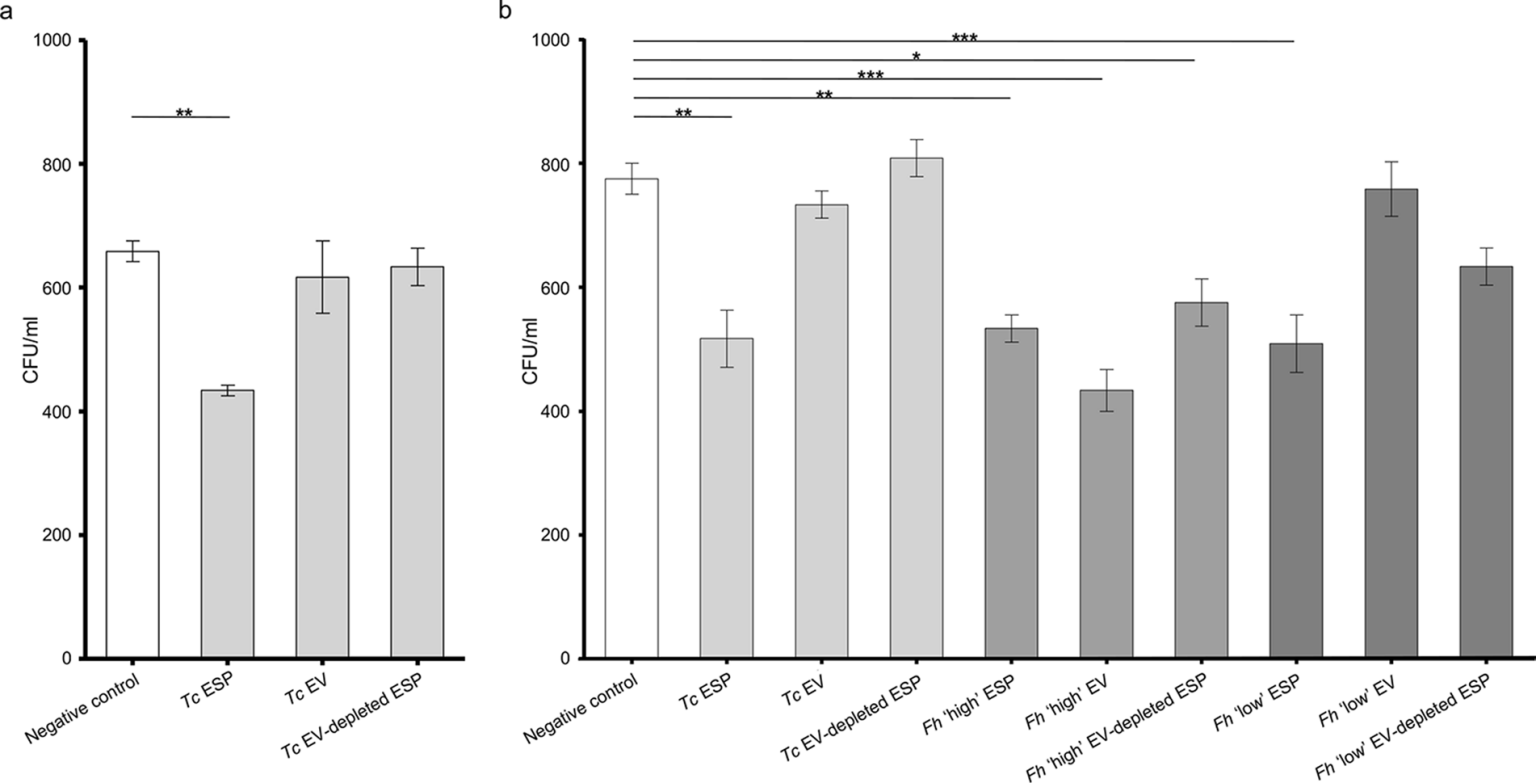
Antibacterial activity of *T. circumcincta* ESP, EVs and EV-depleted ESP.** a** *lux*CDABE-transformed *Escherichia coli* growth following exposure to SEC-purified EVs from ESPs from adult *T. circumcincta*, as well as to *T. circumcincta* whole and EV-depleted ESPs.** b** *lux*CDABE-transformed *E. coli* growth following exposure to SEC-purified EVs from ESPs from adult *T. circumcincta*, as well as to *T. circumcincta* whole and EV-depleted ESPs, alongside SEC-purified EVs from ESPs from adult* Fasciola hepatica* (at ‘high’ and’low’ concentration; see Methods) and *F. hepatica* whole and EV-depleted ESPs (at ‘high’ and ‘low’ concentrations). *P ≤ 0.05, **P ≤ 0.01, ***P ≤ 0.001. Number of replicates (*n*): 3. Error bars represent standard error of the mean. CFU, Colony-forming units;* Fh*,* Fasciola hepatica*;* Tc*, *Teladorsagia circumcincta*

A proof-of-concept experiment was conducted to assess the antibacterial activity of *T. circumcincta* ESPs (at a final concentration of 28 μg/ml), EVs (at a final concentration of 3.65 × 10^8^ particles/ml) and EV-depleted ESPs (at a final concentration of 21 μg/ml) (Fig. 2a). The addition of *T. circumcincta* whole ESPs resulted in a significant reduction of *E. coli* CFU/ml (*P* ≤ 0.05) compared to the negative control (Fig. 2a). Conversely, addition of *T. circumcincta* EVs and EV-depleted ESPs, respectively, did not result in significant reductions of CFU/ml (Fig. 2a). In order to investigate whether the low initial concentrations of SEC-purified EVs and EV-depleted ESP fractions might have impaired the assessment of their antimicrobial activity, we performed an additional proof-of-concept experiment utilising SEC-purified EVs and whole and EV-depleted ESPs from the liver fluke, *F. hepatica*. While no significant reduction in *E. coli* growth was observed when bacteria were exposed to *T. circumcincta* EVs or EV-depleted ESPs (Fig. 2b), significant reductions in *E. coli* CFU/ml were observed following exposure to ‘high’ concentrations of *F. hepatica* SEC-purified EVs (*P* ≤ 0.0001) and to whole (*P* ≤ 0.01) and EV-depleted ESPs (*P* ≤ 0.05) (Fig. 2b). Conversely, exposure of *E. coli* to ‘low’ concentrations of *F. hepatica* SEC-purified EVs and to whole and EV-depleted ESPs yielded results largely consistent with those obtained from *T. circumcincta*, with only *F. hepatica* whole ESPs demonstrating significant antimicrobial activity (*P* ≤ 0.01) (Fig. 2b).

## Discussion

A plethora of studies published over the last decade has pointed toward the occurrence of a complex network of interactions between GI helminths of humans and animals and the resident gut microbiota [38–44]; nevertheless, in spite of this evidence, the exact mechanisms underpinning such interactions have remained largely elusive [15, 45, 46]. In this study, we demonstrated that ESPs from *T. circumcincta*, a globally distributed GI nematode of small ruminants, exert antimicrobial activity in in vitro bacterial growth assays, and identified and characterised several proteins with putative antimicrobial activity that represent key targets of follow-up mechanistic studies of parasite-microbiota relationships. Such putative AMPs were detected both in the EV fraction of *T. circumcincta* ESPs and in EV-depleted ESPs. Amongst the former, we identified a protein sequence sharing significant similarity to an *Ancylostoma*-secreted protein (ASP)-like protein. ASPs are a family of cysteine-rich secreted proteins (CRISPs) belonging to the pathogenesis-related protein superfamily [47] and have been extensively reported as key components of ESPs from a number of helminth species [47–49] (reviewed by [50]); ASPs were also previously described as particularly abundant in EVs from *T. circumcincta* L4s [25]. In spite of their ubiquitous presence and abundance in helminth ESPs, the exact function(s) of parasite ASPs remain(s) largely unknown. However, these proteins are likely to play essential role(s) in host-parasite interactions (reviewed by [50]). For instance, one ASP secreted by the canine hookworm *Ancylostoma caninum* (i.e. ‘neutrophil inhibitor factor’) prevents the release of H_2_O_2_ from activated neutrophils and blocks the adherence of the latter to vascular endothelial cells, thus playing a key immunomodulatory role [51]. Furthermore, two other ASPs secreted by *A. caninum* block the function of integrin receptors on the surface of platelets, inhibiting platelet aggregation and adhesion [52]. In a more recent study, a secreted ASP from *N. americanus*, *Na*-ASP-2, induced neutrophil and monocyte migration and accumulation within host tissue. This pro-inflammatory activity was shown to lead to increased host tissue permeability, which was hypothesised to assist the migration of *N. americanus* larvae through host tissues [53]. Moreover, ASP-1 present in *O. ostertagi* ESPs acts as a protective antigen in vaccine studies [54]. Aside from their likely roles in the infection process [55], ASPs have been speculated to exhibit angiogenesis properties (e.g. in mice infected with the filarial nematode *Onchocerca volvulus* [56]) and protease regulatory activity (in humans infected by *S. mansoni* [57]), thus supporting a diverse array of biological functions for this group of helminth proteins [47]. The likely antimicrobial properties of worm ASPs had been documented in the free-living nematode *Caenorhabditis elegans* (reviewed by [50]); indeed, transcription of the ASP encoding gene, *scl-2*, has been shown to be upregulated in response to infection with Gram-positive *Microbacterium nematophilum* [58]. Transcription of two other ASP-encoding genes, *scl-20* and *scl-27*, was also upregulated in response to infection with multiple bacterial pathogens, including *Pseudomonas aeruginosa* and *M. nematophilum*, thus supporting a role of these proteins in antimicrobial defence [59, 60]. Therefore, a role of EV-associated *T. circumcincta* ASPs as mediators of worm-microbiota interactions in the ruminant host is highly plausible.

Together with ASP homologues, the list of putative AMPs from adult *T. circumcincta* EVs included a histone H4-like protein. Histone proteins H2A, H2B, H3 and H4 make up the ‘nucleosome’, which is defined as the basic repeating structure of chromatin, and is associated with DNA packaging and regulation of gene expression [61, 62]. Histone proteins have previously been identified as cargo in EVs from L4s of *T. circumcincta* [25] as well as from adult *Echinostoma caproni* [63]. Crucially, histone H4 proteins from human sebocytes (i.e. specialised sebum-producing epithelial cells) have been shown to exert significant antibacterial activity against *S. aureus* and *Propionibacterium acnes,* although the exact mechanism(s) underpinning such antimicrobial properties were not fully elucidated [61]. Nevertheless, in another study, histone H2A proteins derived from the skin secretions of *Oncorhynchus mykiss* (rainbow trout) were shown to disrupt bacterial cells by enveloping target bacterial membranes (a process known as ‘carpet mechanism’) and inducing the formation of transient pores which, in addition to leading to membrane disruption, facilitated ingress of H2A proteins in the cell cytosol and/or nucleus and subsequent inhibition of cellular functions [62]. In a previous study, Tagai et al*.* [64] proposed that the antimicrobial properties of human histone proteins are strictly dependent on their amino acid composition, which is classified as either lysine- or arginine-rich. In the latter study, arginine-rich histone H4 proteins were unable to penetrate the bacterial cell membrane, but instead remained associated with the cell surface. Whilst a limited degree of bacterial killing was observed, the antimicrobial properties of H4 were far less potent than those of lysine-rich H2A and H2B histone proteins, which penetrate the cell membrane and induce bacterial cell death by disabling key cellular processes [64]. Based on these findings, it is tempting to speculate that the antimicrobial properties of *T. circumcincta* histone H4-like proteins might be strictly associated with their packaging into EVs, which may allow them to enter bacterial cells following EV internalisation, bind bacterial nucleic acids and induce cellular disruption. Nevertheless, further mechanistic studies are needed to clarify the mechanisms underpinning the interactions between helminth-secreted EVs and bacterial cells residing in the ruminant gut. A thorough characterisation of helminth EVs and their cargo represent the necessary first step towards the characterisation of these interactions.

In a previous proteomic analysis of cargo proteins contained in EVs isolated from L4s of *T. circumcincta*, a saposin-like protein was identified and characterised [25]. Saposins are 8- to 11-kDa nonenzymatic, acidic, heat-stable and cystine-rich proteins containing a characteristic bundle of α-helices that form the ‘saposin fold’ [26, 65]. Saposins are known to play key functions in a multitude of cellular processes, including sphingolipid degradation, lipid antigen presentation, processing of apoptotic bodies and antimicrobial activity [26]. Proteins with antimicrobial activity containing a saposin domain have been identified in both higher and lower eukaryotes [26]. For instance,* Entamoeba histolytica*, an anaerobic parasitic amoebozoan, produces amoebapores which form pores in target bacterial cell membranes, thus causing cell lysis. Also, human granulysin and porcine NK-lysin degrade the integrity of the cell membrane via electroporation, which results in strong antimicrobial activity against bacteria and fungi [26]. Unlike in the study by Tzelos et al*.* [25], protein sequences containing the saposin domain could not be identified amongst cargo proteins contained in adult *T. circumcincta* EVs. The reason for this discrepancy might be linked to fundamental differences between the composition of cargo proteins contained in EVs from larval stages versus adult parasites. Indeed, EV cargo composition between different life-stages of helminth species has been well documented [18, 66, 67]. Furthermore, the low concentration of *T. circumcincta* EVs subjected to proteomics analyses and/or technical differences between methods applied for EV isolation and purification between our study and that by Tzelos et al*.* [25] (i.e. SEC vs ultracentrifugation, respectively), and that might have led to variations between the two datasets, must also be taken into account. Indeed, in a comparative analysis of EV-purification methods from ESPs of *F. hepatica*, SEC yielded EV populations that were smaller in size and less diverse than those isolated by differential centrifugation [28]. Thus, it is plausible that additional proteins with putative antimicrobial activity are contained as cargo in *T. circumcincta* EVs that were not detected in our study, and this possibility merits follow-up investigations.

Amongst the proteins with putative antimicrobial activity detected in EV-depleted ESPs were homologues of a destabilase from the human hookworm, *N. americanus*, and of a ubiquitin from the blood fluke, *S. mansoni*. Destabilases are a family of invertebrate-type lysozymes encoded by the genomes of a broad range of nematode species, including *Brugia malayi, Toxocara canis* and *Trichinella spiralis* [68]. A lysozyme C-1-like protein was also previously detected in EV-depleted ESPs from *T. circumcincta* L4s [25]. The antibacterial properties of lysozymes are well known; these enzymes can cleave the glycosidic bond between N-acetylmuramic acid and N-acetylglucosamine of peptidoglycan, a key component of the bacterial cell wall, resulting in bacterial cell lysis [69]. In helminths, lysozymes are thought to contribute to defence against pathogenic bacteria. For example, expression of the lysozyme-encoding *C. elegans* genes *lys-1*, *lys-7* and *lys-8* is augmented during infection with the Gram-negative *Serratia marcescens* [70]. Worm lysozymes also participate in processes of helminth nutrition, via digestion of microbes colonising the environment where the parasites reside [68]. Of note, as lysozyme-1 proteins were identified amongst molecules isolated from ESPs of the L4 stage of *H. polygyrus*, these enzymes were implicated as likely effectors of parasite-mediated changes in the murine GI microbiome during worm colonisation [71, 72]. It is also worth noting that our proteomics analyses were limited to proteins > 10 kDa and, therefore, the presence of small antimicrobial peptides in EV-depleted ESPs (and EVs) cannot be excluded; in the future, peptidomics approaches will be instrumental to address this outstanding question.

Although the antimicrobial activity of putative AMPs characterised in the present study has not been experimentally validated, the results of our proof-of-principle experiments point toward a significant antibacterial effect of adult *T. circumcincta* ESPs that might be (at least partially) responsible for the alterations in gut microbiome composition that have been previously described in worm-infected animals [12, 13]. Of note, in the present study, no significant antibacterial activity was detected following exposure of *E. coli* to *T. circumcincta* EVs nor EV-depleted ESPs. We hypothesised that this observation might have been linked to the relatively low concentrations of EVs and EV-depleted ESPs that were obtained following the SEC EV-isolation process. In support of this hypothesis, Midha et al. [17] previously observed that the antibacterial activity of ESPs from the porcine roundworm, *A. suum*, was concentration dependent; in the latter study, 125 μg/ml of *A. suum* ESPs was able to induce *E. coli* agglutination, while 65 μg/ml was unable to replicate this effect. In addition, statistically significant *E. coli* biofilm disruption occurred only following exposure of bacteria to high concentrations of ESPs [17]. Our argument is further supported by the observation that *F. hepatica* EVs and EV-depleted ESPs, diluted at a concentration resembling that of the corresponding *T. circumcincta* fractions, ceased to exert antimicrobial activity. While the low concentration of *T. circumcincta* EVs and EV-depleted ESPs likely impaired a thorough assessment of their antimicrobial activity, the detection of homologues of known AMPs in these fractions is indicative of a possible role of these molecules in helminth-microbiome interactions in vivo. Indeed, although the parasite material used in our proof-of-concept experiments was limited, under field conditions, ruminants are likely to be infected with significantly larger numbers of (multiple species of) live parasites, and thus their gut microbiomes are subjected to prolonged exposure to EV-containing ESPs [73–76]. Nevertheless, the occurrence of mechanisms whereby the antimicrobial activity of *T. circumcincta* ESPs is achieved exclusively following exposure of bacteria to whole parasite secretions (rather than individual fractions) cannot be excluded.

## Conclusions

In this study, we show that adult *T. circumcincta* ESPs exert bactericidal and/or bacteriostatic activity in vitro, and have identified molecules within both the EV-rich fraction of ESPs and EV-depleted ESPs with likely antimicrobial properties. Nevertheless, we could not unequivocally attribute such activity to *T. circumcincta* EVs and EV-depleted ESPs. In order to overcome constraints linked to low concentrations of starting material derived from experimental infections, future investigations might wish to focus on the expression of candidate proteins using bacteria, yeast, fungi and/or other systems [77]. Bacterial killing assays could subsequently be performed using recombinant proteins, which would also allow testing of the concentrations required to reproducibly obtain significant inhibition of bacterial growth [17, 25]. Together with saposins, homologues of ASPs, lysozymes and histones, a list of suitable candidate AMPs could be compiled by conducting comparative bioinformatics analyses of helminth genomic, transcriptomic and proteomic datasets, and assigning priority to putative AMPs shared by several worm species. Furthermore, peptidomics may be used to identify small (< 10 kDa) candidate AMPs within *T. circumcincta* ESPs and EVs that could not be detected in the current study. Once the antimicrobial activity of helminth ESP- and EV-associated AMPs is established, follow-up studies might take advantage of current sophisticated in vitro approaches, such as organoids [78] and gut-on-chip systems [79, 80] for mechanistic investigations of the three-way interactions between the worm and its secretions, the host gut epithelium and immune cells, and the gut microbiome. Ultimately, the elucidation of the mechanisms by which helminth parasites interact with the microbial populations inhabiting the GI tract of vertebrate hosts, thus shaping gut microbiota composition and function, will assist the determination of the impact of such changes on the pathophysiology of helminth disease, and pave the way for the development of novel, sustainable strategies for the control of parasite gastroenteritis in ruminant livestock based on the manipulation of the host gut microbiota.

## Supplementary Information

**Additional file 1: Table S1.** Protein sequence data identified from proteomic analyses of adult *T. circumcincta* extracellular vesicles (EVs) and EV-depleted excretory-secretory products (ESPs).

**Additional file 2: Figure S1.** Adult *F. hepatica* extracellular vesicle visual characterisation.

**Additional file 3: Table S2.** Amino acid sequences identified from proteomic analyses of adult *T. circumcincta *extracellular vesicles (EVs) and results of antimicrobial activity prediction analyses.

**Additional file 4:** **Table S3.** List of amino acid sequences identified from adult *T. circumcincta *extracellular vesicle (EV)–depleted excretory-secretory products (ESPs) proteomic analyses and results of antimicrobial activity prediction analyses.

**Additional file 5: Table S4.** Amino acid sequences from adult *T. circumcincta* extracellular vesicles (EV) and EV–depleted excretory-secretory products (ESPs) with putative antimicrobial activity, identified using AMPA.

## Data Availability

The proteomic data generated in this study are available in Additional file 1: Table S1.

## Abbreviations

AMP: Antimicrobial peptide
ASP: *Ancylostoma*-secreted protein
BP: Biological process
CAMP_R3_: Collection of anti-microbial peptide database
CAP: Cysteine-rich secretory proteins, antigen 5, and pathogenesis-related 1 proteins
CC: Cellular component
CFU: Colony-forming units
CNH: Citron homology
CRIB: Cdc42/Rac interactive binding
CRISP: Cysteine-rich secreted protein
ESP: Excretory-secretory product
EV: Extracellular vesicle
FDR: False discovery rate
GI: Gastrointestinal
GO: Gene Ontology
MF: Molecular function
NTA: Nanoparticle tracking analysis
SEC: Size-exclusion chromatography
TEM: Transmission electron microscopy
