# Supplementary figures and images for: Excretory-secretory products from the brown stomach worm, Teladorsagia circumcincta, exert antimicrobial activity in in vitro growth assays
Source: Parasit Vectors. 2022 Oct 2;15:354. doi: 10.1186/s13071-022-05443-z (PMC9528173; doi:10.1186/s13071-022-05443-z)

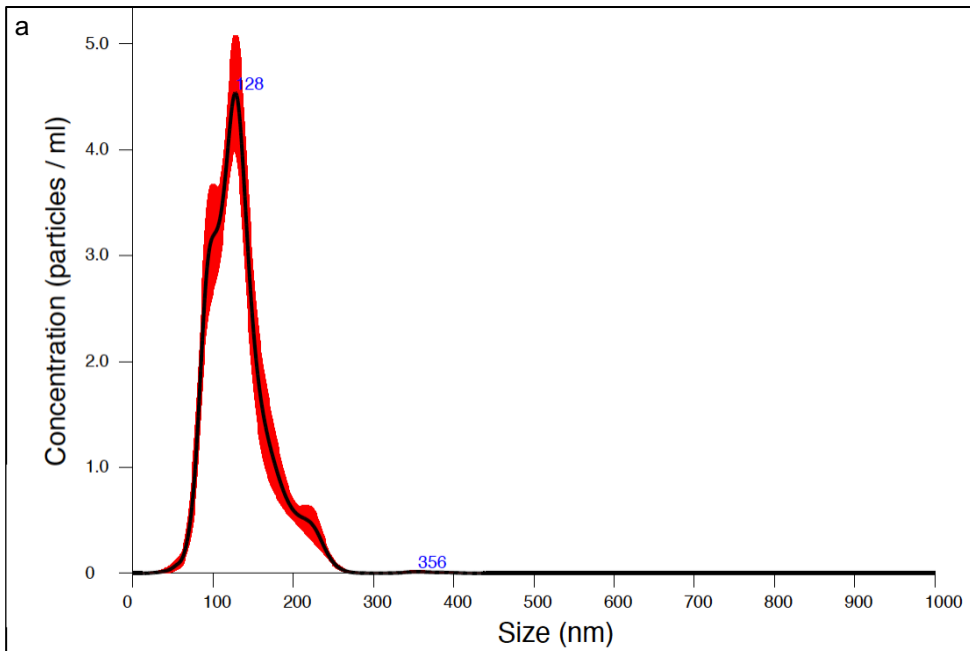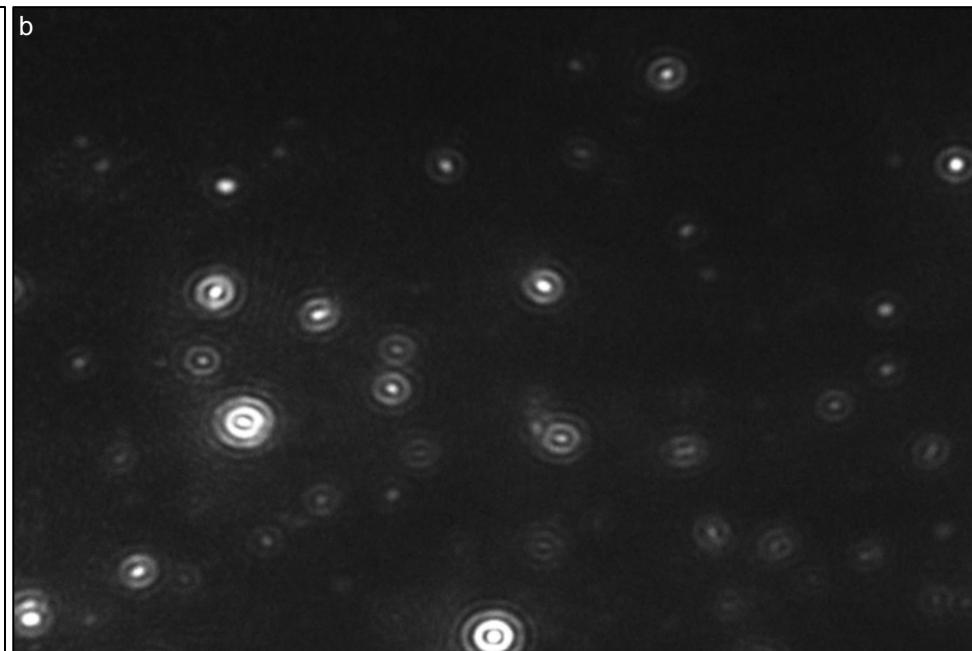

Supplement: Supplementary file 2 — Additional file 2: Figure S1. Adult F. hepatica extracellular vesicle visual characterisation. [file 13071_2022_5443_MOESM2_ESM.pdf]
